# Psychological impact in non-infectious disease specialists who had direct contact with patients with COVID-19

**DOI:** 10.1192/bjo.2020.147

**Published:** 2020-12-07

**Authors:** Tao Liu, Zheng Zheng, Xiaoyan Sha, Huishu Liu, Wenjing Zheng, Huanxing Su, Guiyun Xu, Kuan-Pin Su, Kwok-Fai So, Kangguang Lin

**Affiliations:** Department of Affective Disorders, The Affiliated Brain Hospital of Guangzhou Medical University, China; Department of Obstetrics, Guangzhou Women and Children's Medical Centre, China; Department of Obstetrics, Guangzhou Women and Children's Medical Centre, China; Department of Obstetrics, Guangzhou Women and Children's Medical Centre, China; Department of Affective Disorders, The Affiliated Brain Hospital of Guangzhou Medical University, China; State Key Laboratory of Quality Research in Chinese Medicine, Institute of Chinese Medical Sciences, University of Macau, China; Department of Affective Disorders, The Affiliated Brain Hospital of Guangzhou Medical University, China; Department of Psychiatry & Mind-Body Interface Laboratory (MBI-Lab), China Medical University Hospital, College of Medicine, China Medical University, Taiwan; Department of Affective Disorders, The Affiliated Brain Hospital of Guangzhou Medical University, China; Guangdong-Hong Kong-Macau Institute of CNS Regeneration, Jinan University, Guangzhou, China; and The State Key Laboratory of Brain and Cognitive Sciences and Department of Ophthalmology, University of Hong Kong, Hong Kong, China; Department of Affective Disorders, The Affiliated Brain Hospital of Guangzhou Medical University, China; and Guangdong-Hong Kong-Macau Institute of CNS Regeneration, Jinan University, China

**Keywords:** Mental health, COVID-19, obstetrical staff, depression, anxiety

## Abstract

**Background:**

The coronavirus disease 2019 (COVID-19) outbreak has become a pandemic. Obstetricians and midwives, among other medical staff, are tackling COVID-19 and are under immense psychological stress.

**Aims:**

We aimed to survey the mental health of non-infectious disease specialist staff, specifically obstetricians and midwives, working in officially designated hospitals treating patients with COVID-19.

**Method:**

A nationwide online survey was conducted from 7 March to 17 March 2020 investigating the mental health of obstetricians and midwives (who were not themselves infected with COVID-19) working in hospitals treating patients with COVID-19. We used the 9-item Patient Health Questionnaire (PHQ-9), the 7-item Generalized Anxiety Disorder (GAD-7) scale and the 7-item Insomnia Severity Index (ISI) to assess their symptoms of depression, anxiety and insomnia.

**Results:**

A total of 885 (41.6%), 609 (28.6%) and 729 (34.3%) obstetricians and midwives reported depression (PHQ-9 ≥ 5), anxiety (GAD-7 ≥ 5) and insomnia (ISI ≥ 8), respectively, during the COVID-19 pandemic. Regardless of whether or not they had direct contact with patients with COVID-19, obstetricians and midwives were more likely to report mild and moderate depression and anxiety during the COVID-19 pandemic when compared with before the pandemic. Those who had direct contact with patients with COVID-19 were more likely to report depression and insomnia than those who did not. Those who had sufficient protective equipment or training were less likely to report depression, anxiety and insomnia than those who did not.

**Conclusions:**

Our data suggest that non-infectious disease specialist staff have experienced varying, but increased levels of depression, anxiety and insomnia during this COVID-19 pandemic, which could be reduced by sufficient levels of protective equipment and occupational COVID-19 workplace training.

The coronavirus disease 2019 (COVID-19) outbreak has become a pandemic, resulting in millions of infected individuals and deaths worldwide. Serious concerns have been raised regarding dealing with the psychological issues caused by the disaster, during both the active phase and the extended aftermath. Risk to medical staff is increased because of occupational exposure and the situation is worse in some areas where there is a lack of protective equipment leading to high rates of infection. For instance, in a Wuhan hospital, medical staff (*n* = 40) accounted for 29% of 138 patients with COVID-19.^1^ Apart from infectious disease specialists dealing with COVID-19, other medical staff such as obstetricians and midwives have a high occupational exposure risk.^2,3^ Staff working in obstetrics are often directly exposed to the blood, amniotic fluid and urine of women giving birth and their newborns.

A study evaluating the mental health status of general medical staff during the COVID-19 pandemic has shown that about 23% of medical staff working in the front line had symptoms of anxiety and 27% had a stress response.^4^ To the best of our knowledge, there have been few studies investigating the mental health of staff who were non-infectious disease specialists (such as obstetricians and midwives) fighting against COVID-19, a special group who are at risk of infection.

## Method

### Participants

We conducted a nationwide online survey investigating the mental health of obstetrics staff (obstetricians and midwives) in officially designated hospitals treating patients with suspected or confirmed COVID-19 from 7 March 2020 to 17 March 2020. Investigators invited obstetricians and midwives to participate in the online survey through a platform named ‘Wenjuanxing’.

Inclusion criteria including the following: (a) obstetricians or midwives; (b) aged 16–65 years; (c) women or men; (d) with qualifications to practice as a doctor or a nurse; and (e) working in hospitals treating patients with COVID-19.

Exclusion criteria were: (a) obstetrics staff infected with COVID-19 or with a history of infection with COVID-19; (b) students or trainees; or (c) those who had severe medical conditions that affected their ability to complete the survey.

The study was approved by the Institutional Review Board of Guangzhou Women and Children Medical Care Center (No. 2020-22101). All participants gave electronic informed consent.

### Measurements

#### Mental health

We used widely used self-rated scales to measure depression, anxiety and insomnia. The nine-item Patient Health Questionnaire (PHQ-9),^5^ the seven-item Generalized Anxiety Disorder (GAD-7)^6^ scale and the Insomnia Severity Index (ISI)^7^ were used to assess depression, anxiety and insomnia, respectively. For the PHQ-9 and GAD-7, participants were asked to rate their current status and retrospectively rate their status in the 2 weeks before the COVID-19 epidemic was officially announced by the Chinese media at the end of December 2019. For the ISI, participants were asked to rate their sleeping status for the past month and respectively rate their status in the 1 month before the COVID-19 epidemic was announced by the media at the end of December 2019.

#### Definition: depression, anxiety and insomnia

Based on the PHQ-9 scores, we divided participants into four groups: no depression (scores of 0–4), mild depression (scores of 5–9), moderate depression (scores of 10–14) and severe depression (scores of 15–27).^5^ Based on the GAD-7, participants were divided into four subgroups: no anxiety (scores of 0–4), mild anxiety (scores of 5–9), moderate anxiety (scores of 10–13) and severe anxiety (scores of 14–21).^6^ Based on ISI scores, participants were divided into four subgroups: no insomnia (0–7 points), mild insomnia (8–14 points), moderate insomnia (15–21 points) and severe insomnia (22–28 points).^7^

#### Occupational exposure

We asked participants to answer the following questions in order to assess their occupational exposure.

(a) Have you ever, so far, had direct contact with individuals with suspected or confirmed COVID-19?
(b) Do you think your hospital provides you with sufficient protective equipment for preventing COVID-19?
(c) Do you think your hospital provides you with sufficient professional training in preventing COVID-19?

### Statistical analysis

We used SPSS 26.0 software (SPSS Inc, Chicago, IL, USA) for statistical analyses. Comparisons of the ratios among groups were examined using χ^2^-tests and the Bonferroni method was applied for multiple comparisons. Comparisons of scale scores among three or more groups were analysed by one-way Welch's ANOVA test, and the Games–Howell test was administered for multiple comparisons. Comparisons of scale scores for during and before the COVID-19 epidemic were examined using paired *t*-tests. Comparison of scale scores between two groups was examined using independent sample *t*-tests. Two-tailed significance level was set at *P* < 0.05

## Results

### Participant characteristics

A total of 2259 questionnaires were returned, with 32 (1.4%) rejections and 101 (4.5%) participants from non-obstetric medical staff, resulting in 2126 participants overall being included in our analysis. Of these, 1531 (72%) were working in southern China and the rest were in the northern China. Ages varied from 16 to 65 years old. Women accounted for 97.7% of the sample. There were 770 (36.2%) obstetricians and 1356 (63.8%) midwives.

Obstetricians and midwives aged 31–50 years were more likely to report anxiety (GAD-7 ≥ 5) than those aged 16–30 years (*P* = 0.001). Obstetricians were more likely to report anxiety (GAD-7 ≥ 5) than midwives (*P* = 0.041) (Supplementary Table 1 available at https://doi.org/10.1192/bjo.2020.147).

### Mental health during and before the COVID-19 epidemic

A total of 885 (41.6%), 609 (28.6%) and 729 (34.3%) obstetricians and midwives reported depression (PHQ-9 ≥ 5), anxiety (GAD-7 ≥ 5) and insomnia (ISI ≥ 8), respectively, during the COVID-19 pandemic. Scores on the PHQ-9, GAD-7 and ISI scales in obstetricians and midwives during the COVID-19 pandemic were significantly higher than that before the pandemic (during versus before epidemic scores – PHQ-9: mean 4.41 (s.d. = 4.38) *v.* 3.14 (s.d. = 4.10), *t* = 23.300, *P* < 0.001; GAD-7: mean 3.00 (s.d. = 3.42) *v.* 2.19 (s.d. = 3.42), *t* = 16.196, *P* < 0.001; ISI: mean 6.05 (s.d. = 5.01) *v.* 5.34 (s.d. = 4.86), *t* = 15.381, *P* < 0.001).

As shown in Table 1, we further divided the obstetrical specialists into subgroups based on whether or not they had direct contact with patients with COVID-19 (direct contact group: 371 (17.5%) and no direct contact group 1755 (82.5%)).

**Table 1:** Severity categories of mental health during and before the COVID-19 pandemic

Severity categories	Before the epidemic, *n* (%) (*n* = 2126) (A)	During the epidemic, *n* (%) (*n* = 2126)		*χ*^2^	*P*	Post hoc^a^
		No direct contact with patients with suspected or confirmed COVID-19 (*n* = 1755) (B)	Direct contact with patients with suspected or confirmed COVID-19 (*n* = 371) (C)			
Severity of depression (PHQ-9)				124.139	<0.001	
No depression	1521 (71.5)	1071 (61.0)	170 (45.8)			A > B > C
Mild depression	488 (23.0)	516 (29.4)	146 (39.4)			A < B < C
Moderate depression	74 (3.5)	122 (7.0)	35 (9.4)			A < B, C
Severe depression	43 (2.0)	46 (2.6)	20 (5.4)			A, B < C
Severity of anxiety (GAD-7)				54.210	<0.001	
No anxiety	1664 (78.3)	1279 (72.9)	238 (64.2)			A > B > C
Mild anxiety	394 (18.5)	380 (21.7)	99 (26.7)			A < B, C
Moderate anxiety	27 (1.3)	54 (3.1)	17 (4.6)			A < B, C
Severe anxiety	41 (1.9)	42 (2.4)	17 (4.6)			A < C
Severity of insomnia (ISI)				29.232	<0.001	
No insomnia	1486 (69.9)	1186 (67.6)	211 (56.9)			A, B > C
Mild insomnia	537 (25.3)	453 (25.8)	127 (34.2)			A, B < C
Moderate insomnia	90 (4.2)	104 (5.9)	29 (7.8)			A, B < C
Severe insomnia	13 (0.6)	12 (0.7)	4 (1.1)			

PHQ-9, Patient Health Questionnaire-9; GAD-7, Generalized Anxiety Disorder-7; ISI, Insomnia Severity Index.

a. Bonferroni correction.

There were significant differences in the ratios of severity categories of depression, anxiety and insomnia among the three subgroups (i.e. all participants before the epidemic, and the during with and during without direct contact subgroups) (PHQ-9; χ^2^ = 124.139, *P* < 0.001; GAD-7: χ^2^ = 54.210, *P* < 0.001; ISI: χ^2^ = 29.232, *P* < 0.001). Both the direct contact and no direct contact groups were more likely to reported both mild and moderate depression as well as anxiety during the COVID-19 pandemic when compared with before the pandemic (corrected *Ps* < 0.05).The direct contact group were more likely than the no direct contact group to report severe depression during the COVID-19 pandemic (corrected *P* < 0.05).

More participants in the direct contact group reported severe anxiety during the COVID-19 pandemic compared with before the pandemic (corrected *P* < 0.05). For the measure of insomnia, only the direct contact group were more likely to report mild and moderate insomnia in the pre- and post-pandemic comparison (corrected *Ps* < 0.05). The direct contact group were more likely to reported mild and moderate insomnia than the no direct contact group (corrected *Ps* < 0.05).

As shown in Table 2, there were significant differences in the scores on the PHQ-9, GAD-7 and ISI scales among the three subgroups (all participants before the epidemic, and the during with and during without direct contact subgroups) (PHQ-9: *F* = 59.424, *P* < 0.001; GAD-7: *F* = 32.923, *P* < 0.001; ISI: *F* = 18.675, *P* < 0.001). The direct contact group had higher scores on the PHQ-9, GAD-7 and ISI scales than the no direct contact group (corrected *Ps* < 0.05). Both the direct contact and no direct contact groups had higher scores on the PHQ-9, GAD-7 and ISI scales during the COVID-19 pandemic when compared with scores before the pandemic (corrected *Ps* < 0.05).

**Table 2:** Mean mental health scale scores during and before the COVID-19 pandemic

Scale scores	Before the epidemic (*n* = 2126), mean (s.e.) (A)	During the epidemic, mean (s.e.) (*n* = 2126)		*F*	*P*	Post hoc^a^
		No direct contact with patients with suspected or confirmed COVID-19(*n* = 1755 (B)	Direct contact with patients with suspected or confirmed COVID-19 (*n* = 371) (C)			
PHQ-9 score	3.14 (4.10)	4.15 (4.23)	5.63 (4.84)	59.424	<0.001	A < B < C
GAD-7 score	2.19 (3.42)	2.83 (3.69)	3.82 (4.26)	32.923	<0.001	A < B < C
ISI score	5.34 (4.86)	5.84 (4.92)	7.06 (5.30)	18.675	<0.001	A < B < C

PHQ-9, Patient Health Questionnaire-9; GAD-7, Generalized Anxiety Disorder-7; ISI, Insomnia Severity Index.

a. Games-Howell.

### Mental health by protective measure and training.

As shown in Table 3, we further divided the participants into subgroups based on whether or not they had sufficient protective measures. There were 365 (17.2%) individuals in the sufficient protective measures (SPM) group and 1761 (82.8%) in the insufficient protective measures (IPM) group. Division based on whether or not they had sufficient protective training, placed 185 (8.7%) in the sufficient protective training (SPT) group and 1941 (91.3%) in the insufficient protective training (IPT) group.

**Table 3:** Mental health by protective measure or training

Mental health	Protective measures against COVID-19, *n* (%) (*n* = 2126)		*χ*^2^	*P*	Protective training for COVID-19, *n* (%) (*n* = 2126)		*χ*^2^	*P*
	Sufficient protective measures against COVID-19 (*n* = 1761)	Insufficient protective measures against COVID-19 (*n* = 365)			Sufficient protective training for COVID-19 (*n* = 1941)	Insufficient protective training for COVID-19 (*n* = 185)		
Depression (PHQ-9 ≥ 5)	698 (39.6)	187 (51.2)	16.732	<0.001	787 (40.5)	98 (53.0)	10.734	0.001
Anxiety (GAD-7 ≥ 5)	474 (26.9)	135 (37.0)	14.999	<0.001	540 (27.8)	69 (37.3)	7.421	0.006
Insomnia (ISI ≥ 8)	574 (32.6)	155 (42.5)	13.073	<0.001	646 (33.3)	83 (44.9)	10.057	0.002

PHQ-9, Patient Health Questionnaire-9; GAD-7, Generalized Anxiety Disorder-7; ISI, Insomnia Severity Index.

The SPM and SPT subgroups were less likely to report depression (PHQ-9 ≥ 5), anxiety (GAD-7 ≥ 5) and insomnia (ISI ≥ 8) than the IPM subgroup (χ^2^ = 16.732, *P* < 0.001; χ^2^ = 14.999, *P* < 0.001; χ^2^ = 13.073, *P* < 0.001, respectively) and IPT subgroup (χ^2^ = 10.734, *P* = 0.001; χ^2^ = 7.421, *P* = 0.006; χ^2^ = 10.057, *P* = 0.002, respectively).

As shown in Table 4, the SPM and SPT subgroup had lower scores on the PHQ-9, GAD-7 and ISI scales than the IPM subgroup (*t* = 5.790, *P* = 0.016; *t* = 6.724, *P* < 0.001; *t* = 5.742, *P* < 0.001, respectively) and IPT subgroup (*t* = 5.256, *P* < 0.001; *t* = 4.626, *P* < 0.001; *t* = 4.3042, *P* < 0.001, respectively).

**Table 4:** Mean mental health scale scores by protective measures and training

Scale scores	Protective measures against COVID-19, mean (s.e.) (*n* = 2126)		*t*	*P*	Protective training for COVID-19, mean (s.e.) (*n* = 2126)		*t*	*P*
	Sufficient protective measures against COVID-19 (*n* = 1761)	Insufficient protective measures against COVID-19 (*n* = 365)			Sufficient protective training for COVID-19 (*n* = 1941)	Insufficient protective training for COVID-19 (*n* = 185)		
PHQ-9 score	4.16 (4.26)	5.61 (4.73)	5.790	0.016	4.26 (4.27)	6.02 (5.11)	5.256	<0.001
GAD-7 score	2.75 (3.58)	4.21 (4.59)	6.724	<0.001	2.88 (3.72)	4.23 (4.50)	4.626	<0.001
ISI score	5.77 (4.85)	7.41 (5.53)	5.742	<0.001	5.91 (4.95)	7.56 (5.40)	4.304	<0.001

PHQ-9, Patient Health Questionnaire-9; GAD-7, Generalized Anxiety Disorder-7; ISI, Insomnia Severity Index.

## Discussion

Specialists, including obstetricians and midwives, are on the front line with COVID-19 and are vulnerable to infection. Our survey shows that even obstetricians and midwives who were not infected with COVID-19 reported higher levels of depression, anxiety and insomnia during the pandemic. Obstetricians and midwives who had direct contact with patients with COVID-19 were more likely to report severe depression and anxiety, suggesting they had mood disturbances.

A study by Lai *et al*^8^ surveyed medical staff in China and found that approximately 45% of medical staff reported depression, 40% anxiety and 38% insomnia in Hubei province, and that rates were even higher in the provincial city of Hubei, Wuhan, where COVID-19 was first reported, with the highest infected rate. Prolonged stress and mood disturbances in turn can all cause increased mental burden, disrupt homeostasis and compromise the immune system, which might even further disrupt mood regulation and increase individual's susceptibility to infection.

We observed that mood disturbances and insomnia were more commonly seen in those who had higher occupational exposure. Providing sufficient protective equipment or specific occupational COVID-19 workplace training could help promote better mental health. To help mitigate the stress-related mood disturbance, active behaviours such as light indoor physical exercises and maintaining sleep hygiene should be promoted. Moreover, deep-breathing relaxation and mindfulness meditation techniques could be more widely used by staff to reduce stress and anxiety.^9^ These techniques may be particularly suited for medical professionals for whom physical exercise might not be possible because of the quarantine period during the COVID-19 pandemic.

Our data suggest that during the COVID-19 pandemic obstetricians and midwives have demonstrated high levels of depression, anxiety and insomnia, and the situation is worse in those who had direct contact with patients with COVID-19. Providing sufficient protective equipment and training could help them (re)gain mental well-being.

## Data Availability

The data that support the findings of this study are available from the corresponding author, K.L., upon reasonable request.

## Supplementary material

For supplementary material accompanying this paper visit https://doi.org/10.1192/bjo.2020.147. click here to view supplementary material
