# Supplementary material for: Psychological impact in non-infectious disease specialists who had direct contact with patients with COVID-19
Source: BJPsych Open. 2020 Dec 7;7(1):e8. doi: 10.1192/bjo.2020.147 (PMC7844165; doi:10.1192/bjo.2020.147)
Supplement: Supplementary file 1 [file S2056472420001477sup.zip › S2056472420001477sup001.docx]

| **Supporting Information Table S1** Demographic characteristics and mental health by demographic characteristics | | | | | | | | | | |
| --- | --- | --- | --- | --- | --- | --- | --- | --- | --- | --- |
|  |  |  | Depression(PHQ-9≥5) | |  | Anxiety(GAD-7≥5) | |  | Insomnia(ISI≥8) | |
| Items | Total, No. (%) |  | No. (%) | *p* |  | No. (%) | *p* |  | No. (%) | *p* |
| Age (years) |  |  |  | 0.182 |  |  | 0.001 |  |  | 0.463 |
| 16-30 | 945 (44.4) |  | 407 (43.1) |  |  | 233 (24.7) |  |  | 313 (33.1) |  |
| 31-50 | 1078 (50.7) |  | 443 (41.1) |  |  | 347 (32.2) |  |  | 383 (35.5) |  |
| 50-65 | 102 (4.8) |  | 35 (34.0) |  |  | 29 (28.2) |  |  | 33 (32.0) |  |
| Gender |  |  |  | 0.100 |  |  | 0.758 |  |  | 0.583 |
| Male | 49 (2.3) |  | 26 (53.1) |  |  | 15 (30.6%) |  |  | 15 (30.6) |  |
| Female | 2077 (97.7) |  | 859 (41.4) |  |  | 594 (28.6) |  |  | 714 (34.4) |  |
| Occupational post |  |  |  | 0.132 |  |  | 0.041 |  |  | 0.182 |
| Obstetrical doctor | 770 (36.2) |  | 337 (43.8) |  |  | 241 (31.3) |  |  | 250 (32.5) |  |
| Midwife | 1368 (64.3) |  | 548 (40.4) |  |  | 368 (27.1) |  |  | 479 (35.3) |  |
| Abbreviations: PHQ-9, Patient Health Questionnaire-9; GAD-7, Generalized Anxiety Disorder-7; ISI, Insomnia Severity Index. | | | | | | | | | | |
